# Relationship among weather variation, agricultural production, and migration: A systematic methodological review

**DOI:** 10.1002/hsr2.2002

**Published:** 2024-04-01

**Authors:** Bishwajit Sarker, Masud Alam, Md. Jamal Uddin

**Affiliations:** ^1^ Department of Statistics Shahjalal University of Science and Technology Sylhet Bangladesh; ^2^ Department of Agricultural Statistics Sylhet Agricultural University Sylhet Bangladesh; ^3^ Faculty of Graduate Studies Daffodil International University Dhaka Bangladesh

**Keywords:** agricultural production, climate change, methodology, migration, systematic review

## Abstract

**Background and Aims:**

Two main problems the globe currently facing are migration and weather variation. Weather change has a significant impact on the agricultural industry, which affects the majority of poor people. There is a dearth of adequate methodological documentation when examining the relationship between weather variation, agricultural output, and migration. We aimed to identify methodological reporting difficulties by reviewing the quantitative literature on weather‐related migration through agricultural channels.

**Methods:**

A systematic evaluation was conducted using papers published between January 2010 and June 2022, indexed in the SCOPUS, PUBMED, and Google Scholar databases. Using inclusion/exclusion criteria, we selected 22 original research articles out of 18,929 distinct articles for review, in accordance with the PRISMA guidelines. We extracted data from each study to understand how various concepts, research designs, and investigative techniques influence our understanding of migration patterns related to weather in the agricultural sector.

**Results:**

The majority (64%) of the study's data consisted of time series data. In 50% of the studies, secondary data were used. Additionally, 55% of these studies did not state the sample size. In 40% of the studies, model assumptions were fully adhered to, whereas in 36% of the studies, they were not followed at all. The majority of the articles used the Ordinary Least Squares technique, while about 41% applied the Two‐Stage Least Squares technique. Various tests were conducted across these studies, such as robustness checks (59.1%), endogeneity tests (31.8%), omitted variable bias tests (22.7%), sensitivity analyses (22.7%), and weak instrument tests (13.6%), to name a few. In the research we selected, the methodology section had various shortcomings and lacked organization. Furthermore, the justifications for deviations from model assumptions were unclear, potentially affecting the study outcomes.

**Conclusion:**

This study has important indications for researchers in studying climatic (weather) migration through agricultural channels besides for policymakers by giving a thorough review of the methods and techniques.

## Key points

- Providing narrative insights on how various issues, research designs, and analytical techniques shape our understanding of the association between weather variation and migration via agricultural production.
- Providing a broad overview of the pertinent literature, covering 22 articles, as a complement to earlier studies.
- Our study has important ramifications for researchers studying climatic (weather) migration through agricultural channels as well as for policymakers by giving a thorough review of the methods and techniques currently used in this sector.
- Future studies on weather variation, agricultural production, and migration should use prudent and comparable models that capture whole climatic (weather) impacts on migration through mediating factors like agricultural production.

## 1 INTRODUCTION

The majority of the world's poor reside in Asia and Africa, where agriculture is heavily dependent and changing climatic conditions have a detrimental impact on agriculture.^1^ Even though extreme weather events like floods, cyclones, and droughts have dramatic visual effects, countries and populations that depend on agriculture may suffer more long‐term negative effects on agricultural productivity, production, and risk exposure. Because of landslides, soil degradation, flooding, or salinization, farmers' access to agricultural land may be deteriorating. At the same time, declining farm productivity, fishing, and other related activities could have an impact on food security due to declines in the quality and availability of forests, water, and other ecosystems.^2^

With rising temperatures and an increase in the frequency of extreme events, climate change may stimulate increased migration. Climate change will cause further human displacement in the 21st century.^3^ According to World Bank projections, reduced crop yield due to reduced water availability, storm surges, and increasing sea levels may force between 31 and 143 million people to relocate domestically by 2050.^4^ Numerous studies in the rapidly developing body of literature analyze both the climatic conditions and migration in addition to examining the mechanisms underlying the relationship between the two.^5^, ^6^, ^7^, ^8^, ^9^, ^10^, ^11^, ^12^, ^13^, ^14^, ^15^, ^16^ These studies' findings about the relationship between migration and climate indicate that agricultural incomes are a significant driving force.^7^, ^9^, ^14^ Because of poverty traps, higher temperatures deter migration from low‐income countries, while they promote it in middle‐income countries because of lower agricultural yields. A hump is developed in the link between agricultural income, migration, and temperatures.^8^, ^17^ The majority of migrants live in emerging nations, where agriculture is a major source of work and income in rural areas. On the other hand, climate change largely affects the agricultural sector, especially in developing nations, with significant consequences for food safety, rural life, and productivity.^18^, ^19^

Climate change may result in a drastic loss of farmers' income. It may also discourage the requirements for agricultural laborers and their wages. As, in most rural areas, agriculture is the only major activity, the impact on income from farms pours out to the nonfarm sectors too. Low earnings from agriculture and reduced income opportunities from nonfarming push the victim people to migrate.^20^

A very small number of recent articles thoroughly analyze climate change and the relationship between global migration, highlighting the agricultural channel, using a variety of methods and data sets^7^, ^8^. As a result, the practical question of whether agriculture serves as one of the principal mediators in the relationship between migration and climate change remains unanswered.

This systematic review provides a unique analytical perspective that improves previous methodological studies.^21^, ^22^, ^23^, ^24^, ^25^, ^26^, ^27^ To reflect the wide range and diversity of the quantitative studies, we constructed a systematic screening and selection of studies. Large amounts of data were gathered as part of the analyses, allowing us to depict and contrast various statistical findings from various investigations and traits, comprehend how differences in research methodologies can affect study findings, gain new perspectives on problems, and identify research gaps when it comes to modeling weather variation and climate change, agricultural production, and migration. When analyzing the relationship between several issues, there is no one approach that is always the best; every approach has obvious drawbacks. Numerous approaches, such as mixed, qualitative, and quantitative methodologies, may be appropriate, depending on the situation and research questions.^28^ Our review's primary focus is on quantitative literature. This will not only help scholars better grasp the complexities and interdependencies of modeling, but it will also introduce them to the science of methodological resources and tools that can help them handle some of the more challenging issues.

## 2 METHODOLOGY

### 2.1 Review guideline—PRISMA

The PRISMA standard (Preferred Reporting Items for Systematic Reviews and Meta‐analyses) was developed by Moher et al.^29^ as well, and the procedures referred by Hoque et al.^30^ and Hanvold et al.^31^ are the foundations for this investigation. It can direct researchers as they formulate precise answers to study inquiries. Identification, screening, establishing inclusion and exclusion standards, determining eligibility, quality evaluation, data collection, abstraction, and analysis are all included in the systematic search methods used by Ishtiaque et al.^32^ Our systematic review fully adheres to the PRISMA guidelines (Table 1).

**Table 1:** Reporting checklist for systematic review (with or without a meta‐analysis). Based on the PRISMA guidelines.

		Reporting item	Page number/location
**Title**			
Title	#1	Identify the report as a systematic review	Pg‐1
**Abstract**			
Abstract	#2	Report an abstract addressing each item in the PRISMA 2020 for Abstracts checklist	Pg‐2
**Introduction**			
Background/rationale	#3	Describe the rationale for the review in the context of existing knowledge	Pg‐3
Objectives	#4	Provide an explicit statement of the objective(s) or question(s) the review addresses	Pg‐5
**Methods**			
Eligibility criteria	#5	Specify the inclusion and exclusion criteria for the review and how studies were grouped for the syntheses	pg‐7
Information sources	#6	Specify all databases, registers, websites, organizations, reference lists, and other sources searched or consulted to identify studies. Specify the date when each source was last searched or consulted	Figure 1
Search strategy	#7	Present the full search strategies for all databases, registers, and websites, including any filters and limits used	Figure 1
Selection process	#8	Specify the methods used to decide whether a study met the inclusion criteria of the review, including how many reviewers screened each record and each report retrieved, whether they worked independently, and, if applicable, details of automation tools used in the process	pg‐8
Data collection process	#9	Specify the methods used to collect data from reports, including how many reviewers collected data from each report, whether they worked independently, any processes for obtaining or confirming data from study investigators, and, if applicable, details of automation tools used in the process	pg‐8
Data items	#10a	List and define all outcomes for which data were sought. Specify whether all results that were compatible with each outcome domain in each study were sought (e.g., for all measures, time points, analyses), and, if not, the methods used to decide which results to collect	Supporting documents
Data items	#10b	List and define all other variables for which data were sought (such as participant and intervention characteristics, and funding sources). Describe any assumptions made about any missing or unclear information	Supporting documents
Study risk of bias assessment	#11	Specify the methods used to assess the risk of bias in the included studies, including details of the tool(s) used, how many reviewers assessed each study and whether they worked independently, and, if applicable, details of automation tools used in the process	pg‐8
Effect measures	#12	Specify for each outcome the effect measure(s) (such as risk ratio, mean difference) used in the synthesis or presentation of results	Figure 1
Synthesis methods	#13a	Describe the processes used to decide which studies were eligible for each synthesis (such as tabulating the study intervention characteristics and comparing against the planned groups for each synthesis; item #5)	Figure 1
Synthesis methods	#13b	Describe any methods required to prepare the data for presentation or synthesis, such as handling missing summary statistics or data conversions	Figure 1
Synthesis methods	#13c	Describe any methods used to tabulate or visually display results of individual studies and syntheses	Figure 1
Synthesis methods	#13d	Describe any methods used to synthesize results and provide a rationale for the choice(s). If meta‐analysis was performed, describe the model(s), method(s) to identify the presence and extent of statistical heterogeneity, and software package(s) used	Figure 1
Synthesis methods	#13e	Describe any methods used to explore possible causes of heterogeneity among study results (such as subgroup analysis, and meta‐regression)	Figure 1
Synthesis methods	#13f	Describe any sensitivity analyses conducted to assess robustness of the synthesised results	Figure 1
Reporting bias assessment	#14	Describe any methods used to assess risk of bias due to missing results in a synthesis (arising from reporting biases)	Figure 1
Certainty assessment	#15	Describe any methods used to assess certainty (or confidence) in the body of evidence for an outcome	Figure 1
**Results**			
Study selection	#16a	Describe the results of the search and selection process, from the number of records identified in the search to the number of studies included in the review, ideally using a flow diagram (http://www.prisma-statement.org/PRISMAStatement/FlowDiagram)	Figure 1
Study selection	#16b	Cite studies that might appear to meet the inclusion criteria, but which were excluded, and explain why they were excluded	Figure 1
Study characteristics	#17	Cite each included study and present its characteristics	Supporting documents
Risk of bias in studies	#18	Present assessments of risk of bias for each included study	Table 5
Results of individual studies	#19	For all outcomes, present for each study (a) summary statistics for each group (where appropriate) and (b) an effect estimate and its precision (such as confidence/credible interval), ideally using structured tables or plots	Supporting documents
Results of syntheses	#20a	For each synthesis, briefly summarise the characteristics and risk of bias among contributing studies	Table 5
Results of syntheses	#20b	Present results of all statistical syntheses conducted. If meta‐analysis was done, present for each the summary estimate and its precision (such as confidence/credible interval) and measures of statistical heterogeneity. If comparing groups, describe the direction of the effect	N/A
Results of syntheses	#20c	Present results of all investigations of possible causes of heterogeneity among study results	Supporting documents
Results of syntheses	#20d	Present results of all sensitivity analyses conducted to assess the robustness of the synthesized results	Table 6
Risk of reporting biases in syntheses	#21	Present assessments of risk of bias due to missing results (arising from reporting biases) for each synthesis assessed	Table 5
Certainty of evidence	#22	Present assessments of certainty (or confidence) in the body of evidence for each outcome assessed	Table 5
**Discussion**			
Results in context	#23a	Provide a general interpretation of the results in the context of other evidence	pg‐08
Limitations of included studies	#23b	Discuss any limitations of the evidence included in the review	pg‐12
Limitations of the review methods	#23c	Discuss any limitations of the review processes used	pg‐12
Implications	#23d	Discuss implications of the results for practice, policy, and future research	pg‐12
**Other information**			
Registration and protocol	#24a	Provide registration information for the review, including register name and registration number, or state that the review was not registered	Was not Registered
Registration and protocol	#24b	Indicate where the review protocol can be accessed, or state that a protocol was not prepared	Protocol was not prepared
Registration and protocol	#24c	Describe and explain any amendments to information provided at registration or in the protocol	Protocol was not prepared
Support	#25	Describe sources of financial or nonfinancial support for the review, and the role of the funders or sponsors in the review	No
Competing interests	#26	Declare any competing interests of review authors	No
Availability of data, code, and other materials	#27	Report which of the following are publicly available and where they can be found: template data collection forms; data extracted from included studies; data used for all analyses; analytic code; any other materials used in the review	pg‐13

### 2.2 Approach to finding and digesting literature

**Figure 1:**
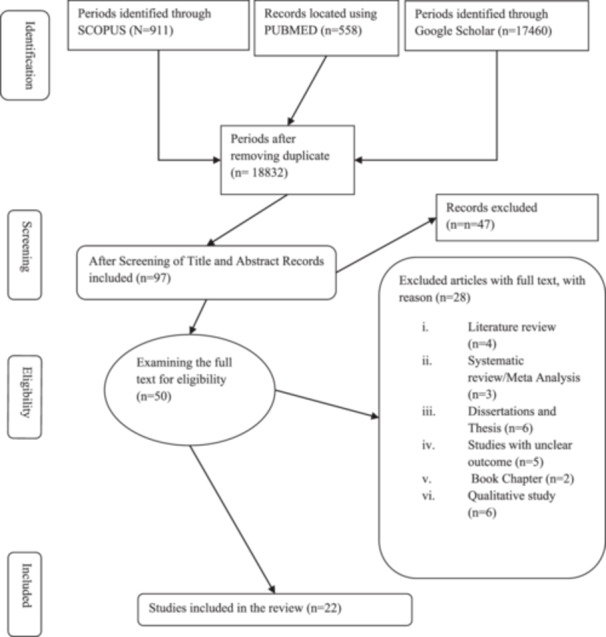
PRISMA flowchart for present review. PRISMA, preferred reporting items for systematic reviews and meta‐analyses.

A methodical approach was used for data searching, which included the structured processes of formulating research questions, identifying potential sources, screening them, assessing their relevance, evaluating their quality, and extracting the necessary information (Figure 1). The sections below provide a detailed explanation of this approach.

#### 2.2.1 Identification

The pertinent information was found by using similar main phrases as well as associated vocabulary,^33^ which were developed based on professional advice and prior research. The most popular databases on climate change for refereed published content were SCOPUS, PUBMED, and Google Scholar, where the majority of the search was concentrated.^34^ Due to their meticulous management and suitability for a methodical evaluation procedure,^35^ as well as for gathering eminent study papers across a range of disciplines,^36^, ^37^ these databases are widely used. To build a database of published articles, the following search terms were used: climate change, and weather variation; labor migration; temperature shocks; rice production; and agricultural production. We chose to employ a Boolean search strategy (Table 2) because of the large number of papers that were produced during the initial search. As a result, to combine the search term and phrase search, each outcome of interest was looked up independently using the “AND” and “OR” operators. Three sources yielded a total of 18,832 articles that were momentarily retrieved.

**Table 2:** Search Boolean databases from SCOPUS, PUBMED, and Google Scholar.

Database	Search term
SCOPUS	Step‐1(1)=(“Climate Change” OR “Weather variation” OR “Temperature Shock”)(2)=(“Labour Migration” OR “Labor Migration”)(3)=(“Agricultural Production” OR “Rice Production”))Step‐2(1) AND (2) AND (3)
PUBMED	((“Climate Change” OR “Weather variation” OR “Temperature Shock”) AND (“Labour Migration” OR “Labor Migration”) AND (“Agricultural Production” OR “ Rice Production”))
Google Scholar	((“Climate Change” OR “Weather variation” OR “Temperature Shock”) AND (“Labour Migration” OR “Labor Migration”) AND (“Agricultural Production” OR “ Rice Production”))

#### 2.2.2 Screening

We supplemented published articles with reports covering various aspects of migration, such as the impact of climate change, underlying motivations, the environment's role, future scenarios, and migration related to agricultural production. Additionally, to gain insights into these dynamics in Bangladesh, we also reviewed literature on the interplay of climate change, agricultural production, and migration from other regions. Two researchers (MJU and MA) independently assessed the full‐text articles for inclusion after screening the titles and abstracts using the eligibility criterion and removing duplicate studies. Any disagreements were resolved with the co‐authors’ assistance. Data were gathered from each of the studies that qualified using a common form. While screening the data, the research question was positioned in the middle. As the impact of climate change on migration through the mediation of different agricultural channels has turned out to be vital issues in recent years, the articles that find the relationship among weather variation, agricultural production, and migration between January 2010 and June 2022 were selected (Table 3). In particular, the articles relating to climate change's impact on migration through agriculture and in the English language were chosen. Review and systematic/meta‐analysis articles, non‐English language articles with paywall restrictions, conference proceedings, dissertations and theses, book chapters, studies with unclear results, and qualitative studies were excluded if they were published before January 1, 2010, though.

**Table 3:** Inclusion and exclusion criteria.

Inclusion criteria	Exclusion criteria
Publications between January 2010 and June 2022	Published before January 1, 2010, though
Articles relating to climate change's impact on migration through agricultural channels	Articles missing any issues
Full‐Text available journal articles	Editorials, letters, and commentaries Case studies, qualitative studies, reports, or case series Literature reviews Guidelines Dissertations and Thesis Book Chapters Full text not available journal Systematic Review/Meta‐Analysis Paywall restrictions Conference proceedings
Pooled estimates studies with clear outcome	Studies without clear outcome
English language articles	Non‐English

A total of 17,460 articles were produced by Google Scholar, 911 by SCOPUS, and 558 by PUBMED's search engine. To guarantee that the publications’ objectives were pertinent to the study's research question, the titles and abstracts of every publication were carefully scrutinized. There were 50 acceptable articles left after 18,929 articles were excluded. Twenty‐eight full‐text articles were excluded, of which 4 were reviews of the literature, 3 were systematic reviews/meta‐analyses, 6 were dissertations and theses, 5 were studies with ambiguous results, 2 were book chapters, 2 were in languages other than English, and 6 were qualitative studies. The rejected articles were deemed unsuitable because they concentrated on how climate change is affecting migration or agriculture without addressing their causal relationships.

#### 2.2.3 Eligibility

Finally, for the systematic review, 22 papers were chosen. Then, all 22 papers were processed, carefully scrutinized for eligibility, and then examined. The entire texts of all 22 publications were then retrieved for quality assessment. The flow diagram in Figure 1 illustrates the processes of a systematic search method.

#### 2.2.4 Quality appraisal

To ensure the quality of the chosen articles and to prevent biases, a quality appraisal was carried out.^32^, ^38^ Two experts (MJU and MA) provided the chosen papers to evaluate the quality of the content. As a result, all 22 papers (Table 4) were extracted for analysis and qualified for the final review using the Cross‐Sectional Studies Critical Appraisal Tool, which examines 8 important aspects, through observational cohort studies (Table 5). A well‐conducted article would receive a minimum score of 1 and a maximum score of 8, with points awarded for adherence to each of those criteria. Two reviewers decided which studies were eligible and of what quality, and they discussed any differences to reach a consensus.

**Table 4:** lists the selected paper.

Sl. no.	Title of the selected paper	Authors	Published year
1.	Climate change and migration: Is agriculture the main channel?	Chiara Falco, Marzio Galeottia, and Alessandro Olper	2019
2.	Linkages among climate change, crop yields, and Mexico–US cross‐border migration	Huaizhang Fengab, Alan B. Kruegeracd, and Michael Oppenheimer	2010
3.	Weather, agriculture and rural migration: evidence from state and district level migration in India	Brinda Viswanathan and K. S. Kavi Kumar	2015
4.	Weather Shocks, Agricultural Production and Migration: Evidence from Tanzania	Zaneta Kubik and Mathilde Maurel	2016
5.	Climate Change, Crop Yields, and Internal Migration in the United States	Shuaizhang Feng, Michael Oppenheimer, and Wolfram Schlenker	2012
6.	The influence of climate variability on internal migration flows in South Africa	Marina Mastrorillo, Rachel Licker, Pratikshya Bohra‐Mishra, Giorgio Fagiolo, Lyndon D. Estes, and Michael Oppenheimer	2016
7.	The migration response to increasing temperatures	Cristina Cattaneoa and Giovanni Perib	2016
8.	Who are the climate migrants and where do they go? Evidence from rural India	Barbora Sedova and Matthias Kalkuhl	2019
9.	Climate Change, Agriculture and Migration: Evidence from Bangladesh	Kazi Iqbal and Paritosh K. Roy	2015
10.	Climate Instability, Urbanisation and International Migration	Mathilde Maurel and Michele Tuccio	2016
11.	Climate variability and international migration: The importance of the agricultural linkage	Ruohong Cai, Shuaizhang Feng, Michael Oppenheimer, and Mariola Pytlikova	2014
12.	Influence of Weather on Temporary and Permanent Migration in Rural India	K. S. Kavi Kumar and Brinda Viswanathan	2013
13.	Migration and Climate Change in Rural Africa	Cristina Cattaneo and Emanuele Massetti	2016
14.	Migration in response to climate change and its impact in China	Yi Sun, Chengjin Xu, Hailing Zhang, and Zheng Wang	2016
15.	Temperature shocks, rice production, and migration in Vietnamese households	Adelaide Baronchelli and Roberto Ricciuti	2022
16.	Climate change, migration, and irrigation	Théo Benonnier, Katrin Millock, and Vis Taraz	2019
17.	Climate Change, Rice Production, and Migration in Vietnamese Households*	Adelaide Baronchelli and Roberto Ricciuti	2017
18.	Climate Change, Agriculture and Migration: Is there a Causal Relationship?	A. Olper, C. Falco, and M. Galeotti	2018
19.	Climate change, agriculture and international migration nexus: African youth perspective	Sosina Bezu Teferi, Demissie Degnet, Abebaw Catherine, Mungai Seble Samuel, Maren Radeny Sophia, and Huyer Dawit Solomon	2020
20.	Climate, Agriculture and Migration: A Critical review of dynamic livelihood changes in the Nepal Tarai	Asheshswor Man Shestha	2017
21.	Climate adaptation by crop migration	Lindsey L. Sloat, Steven J. Davis, James S. Gerber, Frances C. Moore, Deepak K. Ray, Paul C. West, and Nathaniel D. Mueller	2020
22.	Can labor migration help households adapt to climate change? Evidence from four river basins in South Asia	Amina Maharjan, Sabarnee Tuladhar, Abid Hussain, Arabinda Mishra, Suruchi Bhadwal, Sultan Ishaq, Basharat Ahmed Saeed, Ishani Sachdeva, Bashir Ahmad, Jannatul Ferdous, and S. M. Tanvir Hassan	2021

**Table 5:** Quality assessment using the Joanna Briggs Institute (JBI) critical appraisal checklist for analytical cross‐sectional studies.

JBI checklist no. study	JBI critical appraisal checklist for analytical cross‐sectional studies								
	Were the criteria for inclusion in the sample clearly defined?	Were the study subjects and the setting described in detail?	Was the exposure measured in a valid and reliable way?	Were objective, standard criteria used for measurement of the condition?	Were confounding factors identified?	Were strategies to deal with confounding factors stated?	Were the outcomes measured in a valid and reliable way?	Was appropriate statistical analysis used?	Overall comments with JBI quality score
Chiara Falco et al. 2019	Y	Y	N/A	Y	N	N	Y	Y	Included (0.7)
Shuaizhang Feng et al. 2010	Y	Y	N/A	Y	Y	Y	Y	Y	Included (0.9)
Brinda Viswanathan 2015	Y	Y	N/A	Y	N	N	Y	Y	Included (0.7)
Zaneta Kubik et al. 2016	Y	Y	N/A	Y	N	N	Y	Y	Included (0.7)
Shuaizhang Feng 2012	N	Y	N/A	Y	Y	Y	Y	Y	Included (0.9)
Marina Mastrorillo et al. 2016	N	N	N/A	Y	Y	Y	Y	Y	Included (0.8)
Cristina Cattaneoa et al. 2016	Y	Y	N/A	Y	N	N	Y	Y	Included (0.7)
Barbora Sedova et al. 2019	Y	Y	N/A	Y	N	N	Y	Y	Included (0.7)
Kazi Iqbal et al. 2015	N	Y	N/A	Y	N	N	Y	Y	Included (0.7)
Mathilde Maurel et al. 2016	Y	Y	N/A	Y	N	N	Y	Y	Included (0.7)
Ruohong Cai et al. 2014	Y	Y	N/A	Y	N	N	Y	Y	Included (0.7)
K. S. Kavi Kumar et al. 2013	Y	Y	N/A	Y	N	N	Y	Y	Included (0.7)
Cristina Cattaneo et al. 2016	Y	Y	N/A	Y	N	N	Y	Y	Included (0.7)
Yi Sun et al. 2016	Y	Y	N/A	Y	N	N	Y	Y	Included (0.7)
Adelaide Baronchelli et al. 2022	N	Y	N/A	Y	Y	Y	Y	Y	Included (0.8)
Théo Benonnier et al. 2019	Y	Y	N/A	Y	N	N	U	Y	Included
Adelaide Baronchelli et al. 2017	Y	Y	N/A	Y	Y	Y	Y	Y	Included (0.9)
A. Olper et al. 2018	Y	Y	N/A	Y	Y	Y	Y	Y	Included (0.9)
Sosina Bezu et al. 2020	Y	Y	N/A	Y	Y	Y	Y	Y	Included (0.9)
Asheshswor Man Shestha 2017	Y	Y	N/A	U	N	N	U	Y	Included (0.5)
Lindsey L. Sloat et al. 2020	Y	U	N/A	Y	N	N	Y	Y	Included (0.6)
Amina Mahajan et al. 2021	Y	Y	N/A	Y	N	N	Y	Y	Included (0.7)

*Note*: Reviewer: MJU, Date: 15 February 2023; Author: BS, MA Year: 2023. N/A; not applicable. N: No, U: Unclear; Y: Yes.

### 2.3 Analysis of data

Studies based on climate and migration can be broadly divided into macro and micro studies, which deal with migration at the regional or national level and primarily concentrate on individual and household movement. Various research methodologies and data are employed depending on the level of study, ranging from highly localized studies using survey data to global comparisons using country‐level data acquired from administrative systems.^39^ Studies typically address the short‐term migration consequences of weather rather than long‐term climatic changes but have effects on the transferability of the findings. This is because the data is more readily available and has a wide range. However, bodies of research show how short and medium‐term occurrences, like storms, droughts and climate change, are related.^40^, ^41^, ^42^ It is useful for determining the long‐term climate change repercussions.^43^ In our research, we took into account how the weather affects migration, including both extreme and sluggish shifts that can be seen and predicted by climatic trends. By descriptive statistical analysis through Excel 2022 and Statistical Packages for Social Sciences (SPSS 22), the results of all the studies were compiled.

## 3 RESULTS

**Figure 2:**
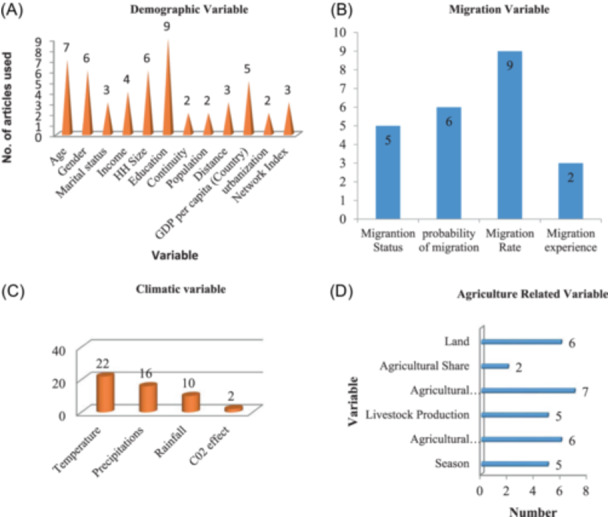
Variables used in different articles are divided into four categories. (A) Demographic variable. (B) Migration variable. (C) Climatic variable. (D) Agriculture‐related variable.

At first, we identified 18,929 distinct articles and reviewed all of these studies. However, 18,810 articles were excluded due to our exclusion criteria, so we only selected 22 articles for further examination. Figure 1 shows the detailed descriptions of the studies. We divided all the variables used in different articles into four (Figure 2A–D) categories, that is, demographic variables, climatic variables, agriculture‐related variables, and migration variables. Age, education, gender, and household sizes were the demographic variables used; Temperature, precipitation, and rainfall were the climatic variables; Agricultural production/output, agricultural productivity, land, and season were agriculture variables; migration status, probability of migration, migration rate was the mostly used in migration variable.

**Figure 3:**
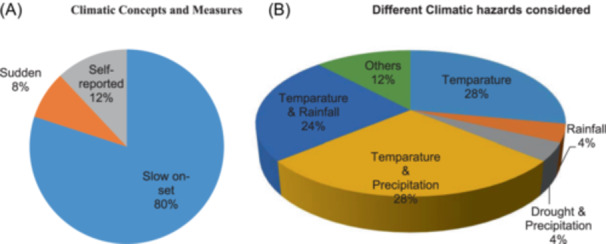
The percentage of research utilizing self‐reported subjective climatic measurements and the proportion of studies concentrating on slow‐onset and rapid climatic occurrences are both shown in Panel A. Slow‐onset events are climatic occurrences that take time to develop, whereas sudden events are sudden occurrences like intense storms, torrential downpours, or flooding. Self‐reported refers to climatic occurrences that survey participants reported. The distribution of research by the various types of climate hazards taken into consideration is shown in Panel B. (A) Climatic concepts and measures. (B) Different climatic hazards considered.

A total of 80% of studies were slow‐onset studies, 8% of studies were on rapid climatic events, and 12% used self‐reported subjective climatic measures (Figure 3A,B).

**Figure 4:**
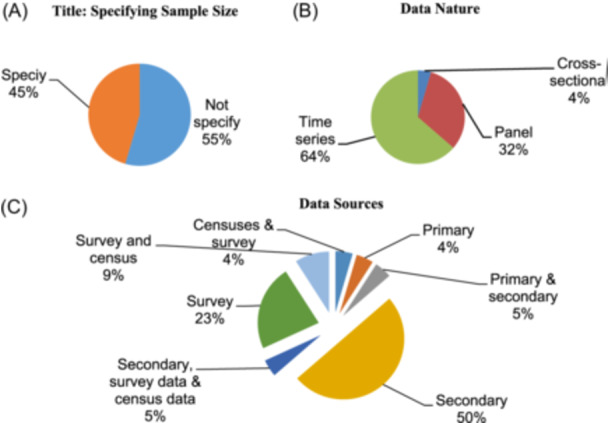
The distributions of reserarch by various types of data (A). Title: Specifying sample size. (B)Data nature. (C)Data sources.

Time‐series data were the type of data (Figure 4B) that was utilized in various publications the most (64%) often. About 50% of studies used secondary sources data (Figure 4C) and 55% of studies did not specify the sample size (Figure 4A).

**Figure 5:**
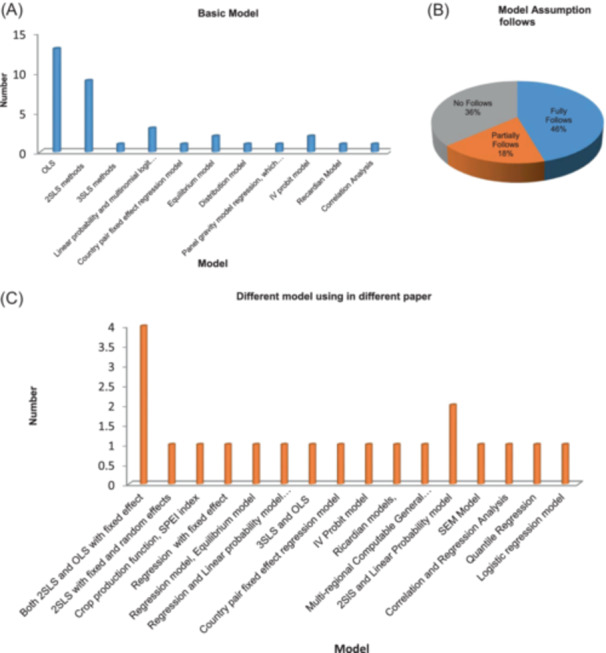
Different models and tests used in different articles with model assumptions followed. (A) Basic model. (B) Model assumption follows. (C) Different models using in different paper.

Different models used by different studies (Figure 5A,C), such as Ordinary Least Square (OLS), Two Stage Least Square (2SLS), Three Stage Least Square (3SLS), Equilibrium model, Linear probability model, Ricardian Model, Country Pair fixed effect model, Panel Gravity model, Instrumental Variable (IV), probit model. Among them, most of the articles used the OLS model and about 41% of studies applied 2SLS methods.

Model assumptions (Figure 5B) fully followed only 40% of studies, where 36% of studies did not follow and 18% partially followed. Different tests (Table 6) were applied by different studies, mostly robustness Check (59.1%), test for endogeneity (31.8%), omitted variable bias test (22.7%), sensitivity analysis (22.7%), test for weak instruments (13.6%), and so on.

**Table 6:** Different test using in different articles.

Test	Test using in articles percentage (using article number/22)*100
Model selection on best‐fit statistics	13.6
Attrition analysis	9.1
Adjusted *R* ^2^	22.7
Dickey–Fuller (DF) test	4.5
Standardized Precipitation‐Evapotranspiration (SPEI) index	4.5
**Omitted variables bias**	22.7
Wald test	4.5
**Test for endogeneity**	31.8
Durbin–Wu–Hausman test	4.5
Hansen J statistics test	9.1
**Test for weak instruments**	13.6
Under‐identification test	4.5
Kleibergen‐Paap Wald F‐statistic	4.5
Anderson‐Rubin (AR) test statistics	4.5
Fractionally re‐sampled Anderson and Rubin (FAR) test	4.5
Cragg–Donald Wald F‐statistic	4.5
**Sensitivity analysis**	22.7
Placebo test	9.1
**Robustness check**	59.1

## 4 DISCUSSION

In our work, we evaluated the effectiveness of the existing models to determine the causal relationship between migration via agricultural productivity and climate change (weather fluctuation). In this review, the majority of the papers employed secondary sources of data, where both census and survey were involved both census and survey data have a limitation when we talk about migration. In general, national censuses are only conducted once every few years (i.e., every 10 years), which may cause them to miss short‐distance and short‐term displacements, such as absences of less than 6 months and displacement within districts or Thana. Moreover, migration linked to climatic shocks and population registries, which are frequently linked to climatic impacts, are underrepresented and may thus go unrecorded. Examples include transitions from rural to urban areas where many residents are not registered with local or national authorities, resulting in discrepancies between the de facto and de jure place of residence.^44^, ^45^ Surveys are a useful tool for analyzing migration, but they are subject to measurement and sampling flaws. The difficulty of choosing a suitable sample size for a sizeable percentage of migrants as uncommon elements in the population, the lack of acceptable sampling frames, and the challenges of locating and identifying migrants pose particular challenges to the sampling of migrants.^46^, ^47^ On the other hand, micro‐level research can offer information on a variety of aspects of migration, including thorough details on the causes, conditions, and effects of movement. They are frequently carried out in the migrant's home country by gathering unofficial data from household members or other proxy respondents, including neighbors, or in the destination area by asking retrospective questions about prior migrations. Both methods of data collection may contain biases.^48^ If an individual or family has relocated more than once in a short period, retrospective inquiries may only provide hazy information about the reasons for the migration and the circumstances under which it took place within a certain time frame. If respondents have trouble recalling the migration process or if it's unclear who is a household member and who is not, utilizing ambiguous data on absent family members in the original locations may also be inaccurate. Also, if a household migrates rather than a single person, there is frequently no one left to provide accurate information on the household's premigration circumstances, the migration triggers, and the household's current location. Any migration research that is conducted only in the regions of origin has this intrinsic limitation, which could result in migrants being underrepresented and undercounted in the sample.^46^

Obtaining our understanding of migratory movements could surely be improved with better climate and migration data. But they arrive with a variety of moral difficulties. The practice of gathering and analyzing migration data through digital trails could have negative effects on the data's security and privacy. Hence, to balance the improvements and the privacy of individuals, a cautiously unbiased method of data collection is necessary.

In our research, we discovered that only a small number of articles tested for the optimum model choice and didn't highlight the rationale for utilizing models. To determine the relationship between climate shocks and migration, various diagnostic techniques might be used. Even though in cross‐sectional analysis, overturn causality is usually not a concern because climatic variables are often calculated in the short run. Yet, the analysis may be biased by omitted variables. This occurs when a variable from the model that is connected to both climatic events and migration is left out, leading to the inclusion of the missing variable(s). For instance, an area's topography or location may influence both its climatic conditions and migration trends^49^, ^50^ (Dell et al., 2014). When analyzing longitudinal panel data, it is advised to employ fixed effects to cope with omitted variables and control missed heterogeneity.^7^, ^51^ It permits the causal interpretation of the response coefficients of the model under these presumptions. Estimates may be skewed by a variety of advanced problems connected to the model's definition. The predictable effects may likely increase due to the correlation between climatic shocks and climate, which would impose a bias in the omitted variable.^21^ Adding possibly influencing control factors that are influenced simultaneously by climate shocks and contain a causal relationship to migration is a common specification problem in the literature. For example, institutional quality, conflict, and poverty are likely influencing aspects of the climatic situation and impact on migration. The assessment of the relevant overall climatic impact on migration would no longer be accurate if these parameters were taken into account in a model; instead, one would only obtain the fractional impact that permeates the mediating channel.^22^, ^50^ While some models with managed mediating variables can provide valuable insight into the mechanisms and channels at play, such models do not concur with concluding about the impact of all climate occurrences on migration. Here, we encourage researchers to use controls that directly address the stated study topic and to omit difficult factors from the analysis, such as agricultural output. It is always advised to employ a model that primarily focuses on the causal relationship between the pertinent climatic shocks as a baseline for model comparisons.^22^ This makes it easier to conduct future meta‐analyses by combining coefficients from several models. Instrumental variable methods, which concentrate on generating a fair assessment of how a mediating pathway affects migration, are a frequent strategy used to examine the causative processes of climatic impacts on migration.^13^, ^52^ In our analysis, around 9 publications each employed 2SLS, where climatic factors are typically used as exogenous variables, referred to as instruments for predicting the mediators in the first stage and the second stage to acquire an objective evaluation of the result of the mediating channel. Strong presumptions underlie the method. First and foremost, the mediator and the instrument must have a close relationship. Second, any channel other than the intermediary under consideration should not have an impact on the migration's outcome (i.e., the exclusion restriction criteria). However, our investigation revealed that a small number of papers did not accurately adhere to the model assumptions.

The cross‐sectional Ricardian technique was created by Mendelsohn et al.^53^ to pinpoint the impact of regional variation in long‐term climatic circumstances. The analysis of time series or longitudinal panel data is another approach frequently employed in the literature to examine the relationship between migration and climate.^54^, ^55^ The gravity model is frequently used when two‐sided global migration is taken into account. OLS allow foran estimate when the migration result contains some zero observations. Negative binomial or Poisson regression models are typically used by researchers when the outcome is zero‐inflated, such as for count data.^56^ If an individual or a household relocated or not, the measure of migration in microstudies is often a binary variable at the individual or household level, logit or probit linear probability models are frequently used in this situation. When distinct destinations can be recognized, multinomial models are utilized.^22^ Most studies include a variety of meteorological variables in their models, which are either monitored repeatedly in several models or all at once in one model.^21^

To compare the coefficients of linear and nonlinear models and to enhance the mediation, researchers might utilize the Durbin‐Wu‐Hausman‐Test or the KHB approach^57^ . In our studies, we also found that Different tests were applied by different studies (Table 6), likely robustness Check, test for endogeneity, sensitivity analysis (22.7%), test for weak instruments (13.6%), and so on.

## 5 CONCLUSION

We discovered that many articles did not provide enough information about their study design, sampling technique, data source, reason of choosing model, assumptions of model, and so on, which made it difficult to assess if the conclusions reached by the researchers were supported by their findings. To translate empirical results into projections, a deeper understanding of how people, households, and communities react to weather variability is required. When it comes to modeling migratory decisions, migration models without a doubt consider climatic factors. Future improvements to the relationship between weather fluctuation and migration through agricultural productivity may greatly benefit from a deeper integration of multiple views and processes across disciplines. Without over‐specifying the model, which takes into account the interdependencies among various weather impacts, we provide an exact and particular model of weather difficulties. It would be an excellent starting point for analyzing the various effects, understanding the degree of correlation among them, and how the model findings would be impacted by them if weather variables were included separately and concurrently in the model. We conclude with three important suggestions for additional investigation.

First, advanced quantitative studies of climate (weather) migration via agricultural channels should endeavor to depict weather, migration, and agricultural data and fit models that reflect and are relevant to the conditions on the ground. Available sources of data and their merits and demerits should be considered and the selection should be confirmed by their quality and research questions obviously at hand. Researchers should illustrate various weather, agricultural, and migration data to validate the derived findings. Using data from digital tracing or machine learning may be a promising technique when data is not available, like migration at short‐distance. Second, controlling for spatial heterogeneity and time trends researchers should use longitudinal models to permit for a causal explanation of climatic (weather) impacts. For spatial and temporal clustering and auto‐correlation Standard errors should be adjusted. The surveillance and analysis of long‐term weather changes become getable if quality and longer time series data are available. Third, while bearing in mind the above features, future studies on weather variation, agricultural production, and migration should use prudent and comparable models that capture whole climatic (weather) impacts on migration through mediating factors like agricultural production. It would also make easy meta‐analyses in the future on the issue that aims to quantify the effects of the climate (weather) on migration, such as the effect of rising temperatures. To ensure complete reproducibility and clarity of the results in this situation, it is essential to keep adequate and complete records of all study phases and methodology decisions. Our study has important ramifications for researchers studying climatic (weather) migration through agricultural channels as well as for policymakers by giving a thorough review of the methods and techniques currently used in this sector.

### 5.1 Limitations

Due to time and resource constraints, only one reviewer was able to screen the title and abstract of the 18,929 pieces of literature that were retrieved, which may have led to a few errors when selecting and retrieving pertinent articles. The reviewer's work was calibrated for reliability by another reviewer during the screening process.

## AUTHOR CONTRIBUTIONS

**Bishwajit Sarker**: Data curation; formal analysis; methodology; writing—original draft; writing—review and editing. **Masud Alam**: Investigation; methodology; supervision; validation; visualization; writing—review and editing. **Md Jamal Uddin**: Conceptualization; investigation; methodology; project administration; supervision; validation; writing—review and editing.

## CONFLICT OF INTEREST STATEMENT

The authors declare no conflict of interest.

## TRANSPARENCY STATEMENT

The lead author Md. Jamal Uddin affirms that this manuscript is an honest, accurate, and transparent account of the study being reported; that no important aspects of the study have been omitted; and that any discrepancies from the study as planned (and, if relevant, registered) have been explained.

## ETHICS STATEMENT

The author affirms that this manuscript is an honest, accurate, and transparent account of the study being reported; that no important aspects of the study have been omitted; and that any discrepancies from the study as planned (and, if relevant, registered) have been explained. All authors have full consent to this article.

## Supporting information

Supporting information.

## Data Availability

The full list of data and the data entries for all included studies are provided in the manuscript as a supplementary file. No additional supporting data is available.
